# In Vitro Synergism of Colistin and N-acetylcysteine against *Stenotrophomonas maltophilia*

**DOI:** 10.3390/antibiotics8030101

**Published:** 2019-07-25

**Authors:** Nagaia Ciacci, Selene Boncompagni, Felice Valzano, Lisa Cariani, Stefano Aliberti, Francesco Blasi, Simona Pollini, Gian Maria Rossolini, Lucia Pallecchi

**Affiliations:** 1 Department of Medical Biotechnologies, University of Siena, 53100 Siena, Italy; 2 Fondazione IRCCS Ca’ Granda Ospedale Maggiore Policlinico, Cystic Fibrosis Microbiology Laboratory, 20122 Milan, Italy; 3 Department of Pathophysiology and Transplantation, University of Milan, 20122 Milan, Italy; 4 Fondazione IRCCS Ca’ Granda Ospedale Maggiore Policlinico, Internal Medicine Department, Respiratory unit and Adult Cystic Fibrosis Center, 20122 Milan, Italy; 5 Department of Experimental and Clinical Medicine, University of Florence, 50134 Florence, Italy; 6 Microbiology and Virology Unit, Florence Careggi University Hospital, 50134 Florence, Italy

**Keywords:** colistin, N-acetylcysteine, *Stenotrophomonas maltophilia*, biofilm

## Abstract

*Stenotrophomonas maltophilia* is an emerging global opportunistic pathogen, responsible for a wide range of human infections, including respiratory tract infections. Intrinsic multidrug resistance and propensity to form biofilms make *S. maltophilia* infections recalcitrant to treatment. Colistin is among the second-line options in case of difficult-to-treat *S. maltophilia* infections, with the advantage of being also administrable by nebulization. We investigated the potential synergism of colistin in combination with N-acetylcysteine (NAC) (a mucolytic agent with antioxidant and anti-inflammatory properties) against *S. maltophilia* grown in planktonic phase and biofilm. Eighteen *S. maltophilia* clinical isolates (comprising three isolates from cystic fibrosis (CF) and two trimethoprim-sulfamethoxazole (SXT)-resistant strains) were included. Checkerboard assays showed a synergism of colistin/NAC combinations against the strains with colistin Minimum Inhibitory Concentration (MIC) >2 µg/mL (*n* = 13), suggesting that NAC could antagonize the mechanisms involved in colistin resistance. Nonetheless, time–kill assays revealed that NAC might potentiate colistin activity also in case of lower colistin MICs. A dose-dependent potentiation of colistin activity by NAC was also clearly observed against *S. maltophilia* biofilms, also at sub-MIC concentrations. Colistin/NAC combinations, at concentrations likely achievable by topical administration, might represent a valid option for the treatment of *S. maltophilia* respiratory infections and should be examined further.

## 1. Introduction

*Stenotrophomonas maltophilia* is an emerging global opportunistic pathogen, responsible for a wide range of human infections, including chronic lung colonization and acute exacerbations in patients affected by chronic respiratory diseases, such as cystic fibrosis (CF), chronic obstructive pulmonary disease (COPD), and bronchiectasis [1]. As reported by the Italian Cystic Fibrosis Registry, *S. maltophilia* is the second most common non-fermenting Gram-negative respiratory pathogen, following *Pseudomonas aeruginosa*, in patients affected by CF, with a prevalence of chronic lung colonization of 4.6% and 4.7% in adult and pediatric patients, respectively [2]. Despite the precise clinical relevance of *S. maltophilia* in CF remains undetermined [3,4], chronic pulmonary colonization by *S. maltophilia* has been recently associated with an increased risk of pulmonary exacerbations requiring intravenous antibiotics, lung transplantation, and death [5,6,7]. Due to intrinsic and acquired multidrug resistance mechanisms and the propensity to grow as biofilm, *S. maltophilia* infections are difficult-to-treat and the therapeutic options are very limited [1,5,8,9,10,11]. Clinical breakpoints for the interpretation of susceptibility testing are available only for trimethoprim-sulfamethoxazole (SXT) (i.e., the first-line treatment option) and few other compounds, namely levofloxacin, some beta-lactams (i.e., ticarcillin-clavulanate and ceftazidime), minocycline, and chloramphenicol [12,13].

In order to find new drugs and their combinations to improve outcomes of difficult-to-treat respiratory tract infections, a renewed interest has been recently focused on topical routes of administration (e.g., inhalation, nebulization, and aerosolization), which allow the achievement of high drug concentrations in the lungs with limited systemic toxicity [14,15].

*N*-acetylcysteine (NAC) is a mucolytic agent commonly administered together with antibiotics for the management of lower respiratory tract infections, especially in patients with chronic respiratory diseases characterized by abundant and/or thick mucus production (i.e., CF, COPD, and bronchiectasis) [16]. In addition, an increasing amount of data points to an intrinsic antimicrobial and antibiofilm activity of NAC against some pathogens, including relevant CF pathogens such as *P. aeruginosa*, *S. maltophilia,* and *Burkholderia cepacia* complex (BCC) [16,17]. Colistin is among the last-resort agents for the treatment of infections caused by multidrug-resistant Gram-negative bacteria, and inhaled colistin (alone or in combination with intravenous colistin) has been increasingly used for the treatment of difficult-to-treat respiratory tract infections, especially in CF [15,18,19,20]. Nonetheless, apart from intrinsic resistance of BCC, colistin is not recommended as first-line treatment option for other relevant CF pathogens (e.g., *S. maltophilia* and *Achromobacter xylosoxidans*), due to lack of clinical breakpoints and high rates of organisms with high Minimum Inhibitory Concentration (MIC) values [21]. Recently, it has been shown that colistin/NAC combinations exert a relevant antimicrobial and antibiofilm synergistic activity against *Acinetobacter baumannii* [22]. In particular, high NAC concentrations (potentially achievable by topical administration) can revert the colistin resistance phenotype in this pathogen [22].

The aim of this study was to investigate the potential synergism of colistin in combination with NAC against *S. maltophilia* strains grown in planktonic phase and in vitro biofilm models, at drugs concentrations likely achievable by topical administration.

## 2. Results

### 2.1. Colistin Susceptibility of S. maltophilia Strains Included in the Study

The study was performed with 18 *S. maltophilia* clinical isolates (including isolates from CF and SXT-resistant strains), which had been previously investigated for NAC susceptibility [17]. Among the strains tested, 13 showed colistin MIC >2 µg/mL (MIC range 4–>256 µg/mL), and for the purposes of this study were categorized as “colistin-resistant” (according to the colistin clinical breakpoints for *P. aeruginosa*) (Table 1) [12,13]. The remaining 5 isolates showed colistin MIC <2 µg/mL (MIC range 0.125–1 µg/mL) and were therefore categorized as “colistin-susceptible” (Table 1). Overall, colistin susceptibility patterns of strains included in this study were consistent with those recently reported in other studies on *S. maltophilia* antibiotic susceptibility [8,23].

### 2.2. In Vitro Synergism of Colistin/NAC Combinations against S. maltophilia Strains Grown in Planktonic Phase

Checkerboard assays showed a notable synergistic activity of colistin/NAC combinations against the 13 colistin-resistant strains (i.e., fractional inhibitory concentration index (FICI) ≤0.5) (Table 2). In particular, a decrease of colistin MICs to ≤2 µg/mL (i.e., the susceptibility breakpoint for *P. aeruginosa*) was observed with 12 strains in the presence of NAC 4 mg/mL and with all strains in the presence of NAC >4 mg/mL (Table 2). The 5 colistin-susceptible strains showed no synergistic effect, although a trend toward colistin MIC decrease was observed in the presence of increasing NAC concentrations (Table 2).

### 2.3. Time-Kill Assays of Colistin/NAC Combinations against Three Selected S. Maltophilia Strains Grown in Planktonic Phase

**Figure 1:**
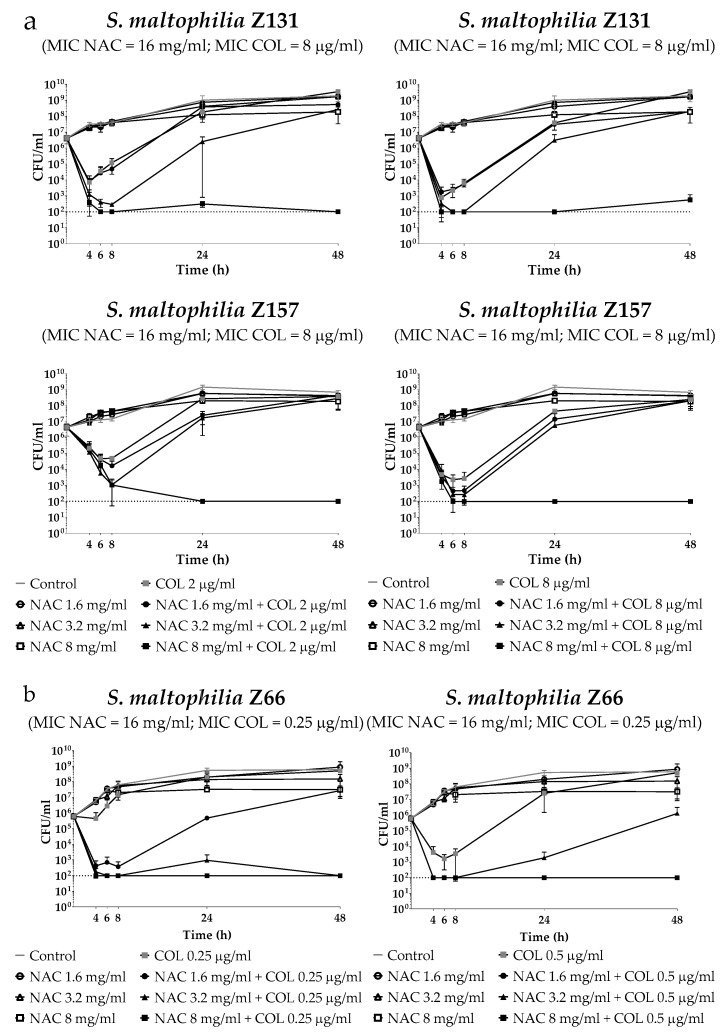
Time–kill assays of colistin/NAC combinations against (**a**) colistin-resistant and (**b**) colistin-susceptible *S. maltophilia* strains. Dotted lines indicate the detection limit (100 CFU/mL). CFU: Colony-forming units.

Time–kill assays were performed with three selected *S. maltophilia* strains, namely Z131 (from bloodstream infection; resistant to SXT, ceftazidime and levofloxacin; colistin MIC = 8 µg/mL), Z157 (from CF; colistin MIC = 8 µg/mL), and Z66 (from lower respiratory tract infection; colistin MIC = 0.25 µg/mL). Colistin and NAC concentrations potentially achievable by topical administration were tested. Results showed a relevant dose-dependent potentiation of colistin activity by NAC, with the three strains investigated (Figure 1a,b). Overall, these data were in accordance with those obtained in checkerboard assays, supporting the notion of synergism of colistin/NAC combinations, and of a possible role of NAC in reverting colistin resistance in *S. maltophilia*.

### 2.4. In Vitro Activity of Colistin/NAC Combinations against S. maltophilia Biofilms

**Figure 2:**
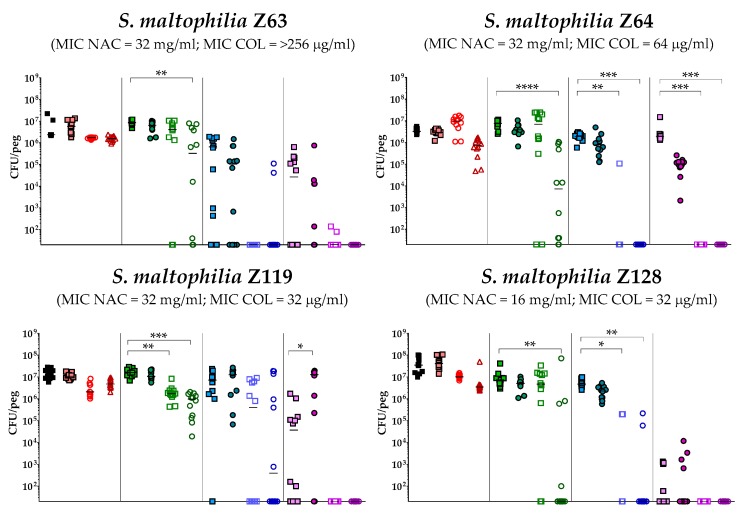

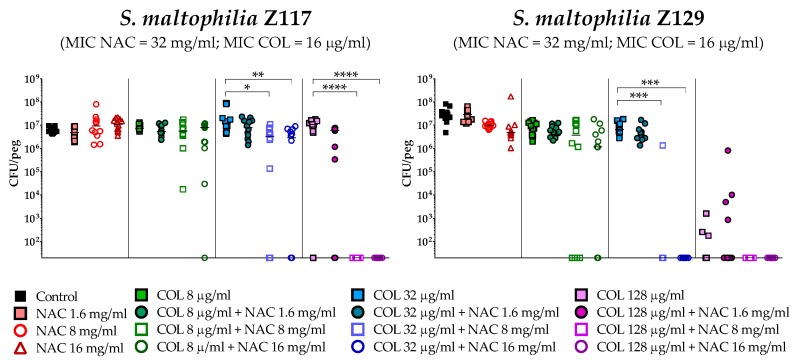
Antibiofilm activity of colistin/NAC combinations against *S. maltophilia* strains with colistin MIC range 16–>256 µg/mL. The x-axis is set at the limit of detection (20 CFU/peg). CFU: Colony-forming units. Each data point represents a replicate, for a total of 12 replicates per condition.

**Figure 3:**
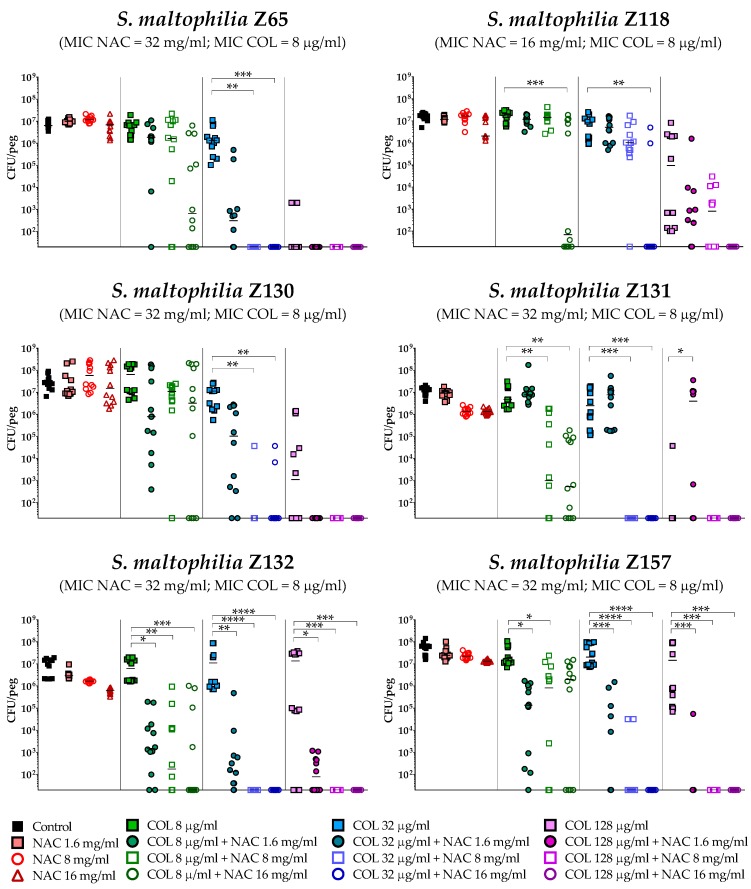
Antibiofilm activity of colistin/NAC combinations against *S. maltophilia* strains with colistin MIC = 8 µg/mL. The x-axis is set at the limit of detection (20 CFU/peg). CFU: Colony-forming units. Each data point represents a replicate, for a total of 12 replicates per condition.

**Figure 4:**
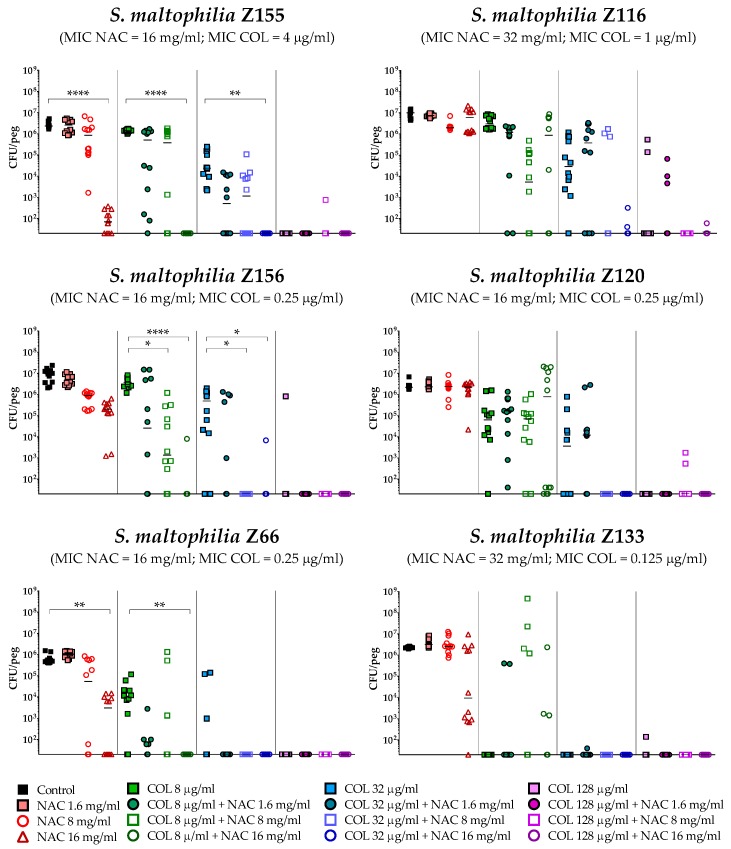
Antibiofilm activity of colistin/NAC combinations against *S. maltophilia* strains with colistin MIC 0.125–4 µg/mL. The x-axis is set at the limit of detection (20 CFU/peg). CFU: Colony-forming units. Each data point represents a replicate, for a total of 12 replicates per condition.

The antibiofilm activity of colistin/NAC combinations was tested against all the 18 *S. maltophilia* strains, using a standardized in vitro biofilm model [24]. Preformed *S. maltophilia* biofilms were exposed to nine different colistin/NAC combinations, and the antibiofilm activity was evaluated by determining the number of viable cells in biofilms treated with colistin/NAC combinations compared to colistin alone. Colistin and NAC concentrations potentially achievable by topical administration were tested. In the in vitro biofilm model adopted, *S. maltophilia* biofilms ranged from 2.5 ± 1.7 × 10^5^ to 1.3 ± 0.4 × 10^7^ colony-forming units (CFU)/peg after 24 h of growth. Overall, a synergism of colistin/NAC combinations was observed with all the colistin-resistant strains, except for strain Z155 (colistin MIC = 4 µg/mL) (Figure 2, Figure 3 and Figure 4). In particular, the combination colistin 8 µg/mL plus NAC 16 mg/mL was synergistic against the majority of colistin-resistant strains (i.e., 7 out of 13 strains) (Figure 2, Figure 3 and Figure 4). *S. maltophilia* Z155 (the only colistin-resistant strain with which no synergism was observed) was extremely susceptible to NAC 16 mg/mL, which alone achieved complete eradication of the in vitro biofilm model (Figure 4). In addition, with two strains (i.e., Z119 and Z131), a paradoxical effect of the combination colistin 128 µg/mL plus NAC 1.6 mg/mL was observed, which will deserve further attention (Figure 2 and Figure 3).

**Figure 5:**
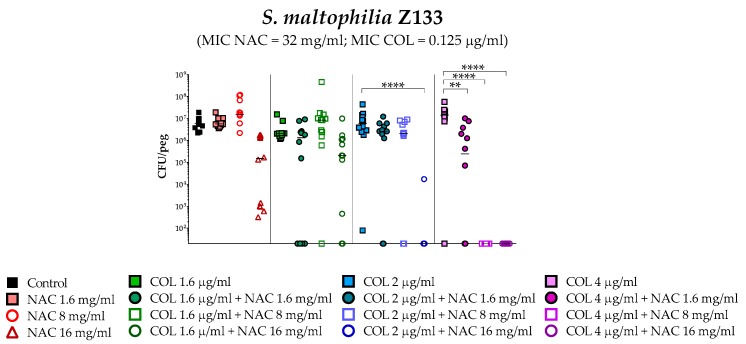
Antibiofilm activity of colistin/NAC combinations against *S. maltophilia* Z133. The x-axis is set at the limit of detection (20 CFU/peg). CFU: Colony-forming units. Each data point represents a replicate, for a total of 12 replicates per condition.

A statistically significant potentiation of colistin activity by NAC was also observed with one of the five colistin-susceptible strains (i.e., Z156) (Figure 4). For the remaining four colistin-susceptible strains, a trend suggesting a potentiation of colistin activity by NAC was observed, even though the results did not reach a statistical significance, likely due to not optimal colistin concentrations tested. In order to partially address this point, strain Z133 was selected and tested also with a lower range of colistin concentrations. Results showed a clear synergism of colistin/NAC combinations also against this strain (Figure 5).

Taken together, these results indicated a NAC-mediated dose-dependent potentiation of the antibiofilm activity of colistin against *S. maltophilia* strains. Biofilm susceptibility to colistin/NAC combinations was anyway strain-dependent and not directly correlated to colistin or NAC MICs.

## 3. Discussion

Colistin/NAC combinations, at the high concentrations potentially achievable by topical administration, have been recently found to exert a relevant synergistic activity against *A. baumannii* grown in planktonic and biofilm phase [22]. In particular, NAC was demonstrated to revert the colistin resistance phenotype in this pathogen and to significantly potentiate colistin antibiofilm activity [22].

Our study demonstrated that the antimicrobial and antibiofilm synergism of colistin/NAC combinations is also exerted against *S. maltophilia*, an emerging global difficult-to-treat opportunistic pathogen, with a relevant role in respiratory tract infections, especially in CF. 

Inhaled colistin has been increasingly used since late 1980s, especially for the treatment of individuals with CF, health care-associated pneumonia, and ventilator-associated pneumonia [15,25,26,27]. Very high colistin concentrations (up to 1137 µg/mL) have been reported in the epithelial lining fluid (ELF) of critically ill patients, after aerosol delivery of 2 million IU (MIU) of colistin methanesulfonate [19,20]. In addition, even higher ELF concentrations are expected to be achieved by using colistin dry powder formulations, which have recently been approved [15,26,28].

Despite inhaled NAC has been used safely for decades as a mucus-dissolving treatment in respiratory diseases associated to abundant and/or thick mucus production (e.g., CF, COPD, bronchiectasis), the actual NAC concentrations achievable in the ELF after topical administration have never been determined. Nonetheless, considering the multiple-dosage regimes of nebulized administration (e.g., 1–10 mL of 200 mg/mL solution every 6–8 h), the higher performance of last-generation nebulizers, and the possibility of direct instillation, topical NAC could reach the ELF concentrations needed for exerting the antimicrobial and antibiofilm potentiation of colistin activity [16,29]. In addition, NAC dry powder formulations have recently been implemented, with the aim of potentiating the penetration through the respiratory mucus of inhaled antibiotics (i.e., clarithromycin and fluoroquinolones) [30,31].

Colistin has gained a renewed interest only in the last years as salvage therapy for the treatment of infections caused by multidrug-resistant Gram-negative pathogens, and many aspects concerning the molecular mechanisms of colistin bactericidal activity and acquired resistance remain still scarcely known [32,33]. Apart from the well described primary mechanism of action (i.e., interaction with lipid A of lipopolysaccharide followed by bacterial membranes derangement), alternative secondary antibacterial mechanisms have been proposed, including inhibition of NDH-2 respiratory chain enzymes (i.e., type II NADH-quinone oxidoreductases) located in the plasma membrane [33,34,35]. In support to the existence of alternative secondary mechanisms of colistin, plasma membrane disruption and oxidative damage have been demonstrated to have a role in colistin bactericidal activity against some Gram-positive bacteria, which lack the primary colistin molecular target (i.e., lipid A of lipopolysaccharide) [36].

Similarly, no solid data are available on the molecular mechanisms accounting for the intrinsic antimicrobial and antibiofilm activity of NAC, which most likely is multifactorial [16]. NAC has been hypothesized to exert its intrinsic antimicrobial activity by competitive inhibition of cysteine utilization, reaction of the NAC sulfhydryl group with bacterial proteins, and perturbation of the intracellular redox equilibrium with potential indirect effects on cell metabolism and intracellular signal transduction pathways [16]. The antibiofilm activity of NAC could be related either to perturbation of microbial physiology (e.g., responsible for inhibition of biofilm formation and/or induction of biofilm disruption), or to a direct destabilization of biofilm matrix architecture (e.g., by chelation of calcium and magnesium or interaction with crucial components in the matrix) [16].

The reasons accounting for the antimicrobial and antibiofilm synergism of colistin/NAC combinations are not easy to be hypothesized due to the relevant knowledge gaps on the mechanisms of action of both compounds. In this perspective, understanding the mechanisms of such a synergism would be relevant not only for optimization of clinical applications but also for drug discovery purposes (e.g., new molecular targets for antibiotic drugs, new compounds able to potentiate colistin activity).

Based on the in vitro evidence of potentiation of colistin antimicrobial and antibiofilm activity by NAC, in vivo animal models to evaluate the potential clinical relevance of topical colistin/NAC combinations are warranted.

## 4. Materials and Methods 

### 4.1. Bacterial Strains Tested

A total of 18 *S. maltophilia* clinical isolates were investigated, including isolates from CF and SXT-resistant strains (Table 1). The strains were the same as in a previous study, aimed at investigating the antimicrobial and antibiofilm activity of NAC against *S. maltophilia* [17]. Colistin (Applichem, Darmstadt, Germany) MICs were determined using the reference broth microdilution method [37]. For the purposes of this study, *S. maltophilia* strains were categorized as susceptible or resistant to colistin based on clinical breakpoints available for *P. aeruginosa* (MIC <2 µg/mL, susceptible; MIC >2 µg/mL resistant) [12,13].

### 4.2. Preparation of NAC-Containing Medium

NAC stock solutions (100 mg/mL) were prepared immediately before use. NAC powder (Zambon, Bresso, Italy) was dissolved in sterile double-distilled water, pH was adjusted at 6.5–6.8 with NaOH, and the solution was filtered through a 0.22-μm membrane filter. All experiments were performed in cation-adjusted Mueller-Hinton broth (CAMHB; Becton Dickinson, Milan, Italy), starting from an appropriately concentrated medium in order to avoid broth dilution when testing high NAC concentrations.

### 4.3. Checkerboard Assays

The potential synergism of colistin/NAC combinations was investigated by checkerboard assay as described previously [38]. The ranges of colistin concentrations tested were 0.003–4 µg/mL and 0.25–256 µg/mL for colistin-susceptible and colistin-resistant strains, respectively. Considering the high drug concentrations potentially achievable by topical administration, the range of NAC concentrations tested was 0.5–32 mg/mL for all strains [16,19]. FICIs values were interpreted as follows: ≤0.5, synergy; >0.5–≤1, partial synergism; >1–4.0, no interaction; >4.0, antagonism.

### 4.4. Time–Kill Assays of Colistin/NAC Combinations Against Planktonic Cultures

Time–Kill assays were performed according to CLSI guidelines [39], with three selected *S. maltophilia* strains: Z131 (from bloodstream infection; resistant to SXT, ceftazidime and levofloxacin; colistin MIC = 8 µg/mL), Z157 (from CF; colistin MIC = 8 µg/mL), and Z66 (from lower respiratory tract infection; colistin MIC = 0.25 µg/mL) (Table 1). Two colistin concentrations (i.e., 2 and 8 µg/mL for colistin-resistant strains; 0.25 and 0.5 µg/mL for colistin-susceptible strains) and three NAC concentrations (i.e., 1.6, 3.2, and 8 mg/mL, corresponding to 0.1× MIC, 0.2× MIC, and 0.5× MIC, respectively, for the selected strains) were tested alone and in combination. Viable cell counts were performed at the beginning of the experiment and after 2, 4, 6, 8, 24, and 48 h of exposure (detection limit, 100 CFU/mL). Data were obtained from at least two independent experiments, with two replicates per condition per experiment.

### 4.5. In Vitro Biofilm Susceptibility Testing

The potential antibiofilm synergism of colistin/NAC combinations was investigated using the Nunc-TSP lid system (Thermo Fisher Scientific, Waltham, MA, USA), as described previously [24]. Briefly, biofilms were grown for 24 h in CAMHB at 35 °C, static conditions. Preformed biofilms were then exposed to three concentrations of colistin (i.e., 8, 32, and 128 µg/mL) and NAC (i.e., 1.6, 8. and 16 mg/mL), alone or in combination. After 24 h of exposure (i.e., 35 °C, static conditions), loosely attached bacteria were removed by two 1-minute washes with 200 µL of phosphate-buffered saline (PBS) (Sigma Aldrich, Milan, Italy). Biofilms were then subjected to 30-minutes sonication (Elma Transsonic T 460, Singen, Germany) in 200 µL of tryptic soy broth (TSB) (Oxoid, Milan, Italy) supplemented with 0.1% Tween 20 (Sigma Aldrich) (i.e., the recovery medium) to remove sessile cells. Mean viable cell counts per peg (CFU/peg) were determined by plating 10 µL of appropriate dilutions of the recovery medium onto tryptic soy agar (TSA) (Oxoid) plates and incubating for 24 h at 35 °C (detection limit, 20 CFU/peg). Colony count was also repeated after 48 h of incubation. Data were obtained in at least two independent experiments, with at least six replicates per condition per experiment.

### 4.6. Statistical Analysis

Statistical analysis was performed using GraphPad Prism version 6.0 (San Diego, CA, USA). D’Agostino-Pearson and Shapiro-Wilk normality tests were applied. Multiple comparison tests were performed by Kruskal-Wallis test with Dunn’s correction.

## 5. Conclusions

In conclusion, we demonstrated a relevant in vitro antimicrobial and antibiofilm activity of colistin/NAC combinations (at the high concentrations likely achievable by topical administration) against *S. maltophilia*, an emerging global difficult-to-treat opportunistic pathogen, with an important role also in CF.

Further studies are needed to understand the molecular bases of such a synergism and to evaluate the potential clinical relevance of colistin/NAC topical formulations.

## 6. Patents

International patent application No. WO2018/154091.

## Figures and Tables

**Table 1:** Main features of the 18 *S. maltophilia* clinical isolates investigated in this study.

Strain	MLST	Origin	Antibiotics				
			MIC (μg/mL)				
			COL	SXT	CAZ	LVX	MIN
Z63	NA	BSI	>256	0.5	2	≤0.25	0.125
Z64	NA	BSI	64	2	64	2	2
Z119	NA	LRTI	32	0.5	32	2	0.5
Z128	NA	LRTI	32	≤0.25	4	1	0.25
Z117	NA	LRTI	16	0.5	64	0.5	0.25
Z129	NA	LRTI	16	≤0.25	4	1	0.25
Z65	NA	IAI	8	1	64	2	1
Z118	ST162	LRTI	8	0.5	8	2	0.25
Z130	NA	IAI	8	0.5	16	16	2
Z131	NA	BSI	8	>8	64	32	1
Z132	NA	LRTI	8	1	2	16	1
Z157	NA	CF	8	0.5	4	2	1
Z155	ST335	CF	4	>8	32	4	2
Z116	NA	LRTI	1	0.5	16	2	0.25
Z156	NA	CF	0.25	1	16	2	0.25
Z120	ST334	LRTI	0.25	0.5	32	1	0.5
Z66	NA	LRTI	0.25	0.5	≤1	1	0.25
Z133	NA	LRTI	0.125	1	2	1	0.25

MLST, multi locus sequence type; NA, not available; BSI, bloodstream infection; LRTI, lower respiratory tract infection; IAI, intra-abdominal infection; CF, cystic fibrosis; COL, colistin (breakpoint not available); SXT, trimethoprim-sulfamethoxazole (S ≤2/38, R ≥4/76 μg/mL); CAZ, ceftazidime (S ≤8, I = 16, R ≥16 μg/mL); LVX, levofloxacin (S ≤2, I = 4, R ≥8 μg/mL); MIN, minocycline (S ≤4, I = 8, R ≥16 μg/mL). S, susceptible; I, intermediate; R, resistant [13].

**Table 2:** Colistin MICs (µg/mL) and corresponding fractional inhibitory concentration indices (FICIs) in the presence of increasing N-acetylcysteine (NAC) concentrations for the 18 *S. maltophilia* clinical isolates investigated in this study. MIC and FICI values corresponding to synergism are shown with grey shading.

Strain	MIC		NAC Concentrations (mg/mL)													
	NAC (mg/mL)	COL (µg/mL)	0.5		1		2		4		8		16		32	
			COL (µg/mL)	FICI	COL (µg/mL)	FICI	COL (µg/mL)	FICI	COL (µg/mL)	FICI	COL (µg/mL)	FICI	COL (µg/mL)	FICI	COL (µg/mL)	FICI
Z63	32	>256	32	0.14	16	0.09	2	0.07	1	0.13	0.5	0.25	≤0.25	0.50	≤0.25	1.00
Z64	32	64	64	0.02	32	0.53	16	0.31	1	0.14	1	0.27	0.5	0.51	0.25	1.00
Z119	32	32	32	1.02	16	0.53	8	0.31	4	0.25	1	0.28	1	0.53	≤0.25	1.01
Z128	16	32	32	1.03	32	1.06	16	0.63	2	0.31	1	0.53	≤0.25	1.01	≤0.25	2.01
Z117	32	16	32	2.02	32	2.03	16	1.06	1	0.19	1	0.31	≤0.25	0.52	≤0.25	1.02
Z129	32	16	32	2.02	16	1.03	16	1.06	2	0.25	1	0.31	0.5	0.53	≤0.25	1.02
Z65	32	8	16	2.02	8	1.03	4	0.56	1	0.25	≤0.25	0.28	≤0.25	0.53	≤0.25	1.03
Z118	16	8	2	0.28	2	0.31	2	0.38	1	0.38	≤0.25	0.53	≤0.25	1.03	≤0.25	2.03
Z130	32	8	16	2.02	8	1.03	2	0.31	1	0.25	1	0.38	0.5	0.56	≤0.25	1.03
Z131	32	8	16	2.02	8	1.03	4	0.56	2	0.38	1	0.38	0.5	0.56	≤0.25	1.03
Z132	32	8	16	2.02	8	1.03	2	0.31	1	0.25	1	0.38	0.5	0.56	≤0.25	1.03
Z157	32	8	32	4.02	32	4.03	8	1.06	2	0.38	1	0.38	1	0.63	≤0.25	1.03
Z155	16	4	4	1.03	4	1.06	2	0.63	1	0.50	1	0.75	≤0.25	1.06	≤0.25	2.06
Z116	32	1	1	1.02	1	1.03	1	1.06	1	1.13	0.5	0.75	0.25	0.75	≤0.003	1.00
Z156	16	0.25	0.5	2.03	0.5	2.06	0.25	1.13	0.25	1.25	0.06	0.75	≤0.003	1.02	≤0.003	2.02
Z120	16	0.25	0.5	2.03	0.5	2.06	0.25	1.13	0.25	1.25	0.06	0.75	≤0.003	1.02	≤0.003	2.02
Z66	16	0.25	0.25	1.03	0.25	1.06	0.25	1.13	0.125	0.75	0.125	1.00	≤0.003	1.02	≤0.003	2.02
Z133	32	0.125	0.25	2.03	0.25	2.06	0.125	1.13	0.125	1.25	0.125	1.50	≤0.003	1.03	≤0.003	2.03
